# Preoperative Liver Function Test Abnormalities Were Associated With Short-Term and Long-Term Prognosis in Cardiac Surgery Patients Without Liver Disease

**DOI:** 10.3389/fcvm.2021.772430

**Published:** 2021-11-01

**Authors:** Liqun Shang, Yuanhan Ao, Linhua Lv, Lihua Lv, Yubi Zhang, Jian Hou, Jianping Yao, Zhongkai Wu

**Affiliations:** ^1^Department of Cardiac Surgery, The First Affiliated Hospital of Sun Yat-sen University, Guangzhou, China; ^2^NHC Key Laboratory of Assisted Circulation, Sun Yat-sen University, Guangzhou, China; ^3^Department of Pharmacy, Shaoyang University, Shaoyang, China; ^4^Department of Hematology Oncology, Shaoyang Central Hospital, Shaoyang, China

**Keywords:** preoperative liver function tests, cardiac surgery, short-term prognosis, long-term prognosis, hospital mortality, 90-day mortality

## Abstract

**Aims:** To explore the value of preoperative liver function tests (LFTs) for the prognosis of cardiac surgery patients without liver disease.

**Methods:** The Medical Information Mart for Intensive Care III (MIMIC-III) database was used to extract the clinical data. Adult cardiac patients (≥18 years) without liver disease in the database were enrolled. The association of LFTs with the time of hospital stay and ICU stay was analyzed with the Spearman correlation. Survival curves were estimated using the Kaplan-Meier method and compared by the log-rank test. Multivariable logistic regression was used to identify LFTs that were independent prognostic factors of mortality.

**Results:** A total of 2,565 patients were enrolled in this study. Albumin (ALB) was negatively associated with the time of hospital stay and ICU stay, while alanine transaminase (ALT), aspartate aminotransferase (AST), and total bilirubin were positively associated with the time of hospital stay and ICU stay (all *p* < 0.001). Abnormal ALB, ALT, AST, and total bilirubin were associated with lower 90-day and 4-year survival (all *p* < 0.001) and could be used as independent risk factors for hospital mortality and 90-day mortality. However, only ALB and total bilirubin were independent risk factors for 4-year mortality.

**Conclusion:** Preoperative LFT abnormalities were associated with short-term and long-term prognosis of cardiac surgery patients without liver disease.

## Introduction

One of the risks of cardiopulmonary bypass (CPB) surgery is to cause postoperative organ dysfunction which may affect the prognosis of patients (1). Approximately 10% of high-risk cardiac surgery patients undergoing CPB can experience liver damage, which directly affects the morbidity and mortality of patients (2). Transient but not permanent abnormal liver function tests (LFTs) after coronary artery bypass graft surgery are possible due to decreased hepatic flow, hypoxia, or pump-induced inflammation (3). Moreover, the severity of liver disease and the degree of abnormality of liver function indicators significantly affect the prognosis of cardiac surgery (4, 5). However, LFTs are very complicated in cardiac surgery patients because not only the operation but also heart disease itself can cause abnormalities.

Abnormal liver function is a common manifestation of heart failure (6, 7) and can be related to a worse prognosis. The detrimental effects of heart failure may be associated with liver congestion secondary to volume and pressure overload, decreased cardiac output, and liver hypoperfusion leading to hypoxic injury (6). Although a variety of cardiohepatic syndromes are recognized by clinicians, there are few investigations of the associations of preoperative LFT abnormalities with short-term and long-term prognosis in cardiac surgery patients.

In this study, we tried to explore the association between preoperative LFTs and the length of hospital and ICU stays, hospital mortality, 90-day mortality, and 4-year mortality of cardiac surgery patients without liver disease.

## Methods

### Data Source

This study was a retrospective cohort study and data were obtained from a freely accessible critical care database, named Medical Information Mart for Intensive Care III (MIMIC-III). The database consists of clinical data of patients who stayed in the ICU of Beth Israel Deaconess Medical Center between 2001 and 2012 (8). After online training under the Collaborative Institutional Training Initiative (CITI) program of the National Institutes of Health (NIH), the right to access the database and acquire the data was approved by the institutional review boards of the Massachusetts Institute of Technology (Cambridge, MA, USA) (Record ID 41268972). As all the data have been de-identified, informed consent was not required.

### Patient Selection

From all patients in the MIMIC-III database, patients were included as follows: (1) patients who underwent on-pump cardiac surgery; (2) those without a history of primary significant liver disease or acute hepatic failure; (3) those older than 18 years; and (4) preoperative routine blood examination was performed within 24 h after admission.

### Data Extraction

All clinical data were queried and extracted by using the Structured Query Language (SQL), and pgAdmin4 was used as the administrative platform for PostgreSQL. The extracted data mainly comprised demographics (age and sex), vital signs [diastolic blood pressure (DBP), heart rate (HR), respiratory rate (RR), systolic blood pressure (SBP), percutaneous oxygen saturation (SpO_2_), and temperature], comorbidities (congestive heart failure, valvular disease, cardiac arrhythmias, hypertension, chronic pulmonary disease, pulmonary circulation disorder, peripheral vascular disease, uncomplicated diabetes, complicated diabetes, liver disease and renal failure), laboratory parameters [peripheral white blood cell count (WBC), serum potassium, serum sodium, platelet count, serum glucose, blood urea nitrogen (BUN), serum creatinine, blood albumin (ALB), blood alanine transaminase (ALT), blood aspartate transaminase (AST), and blood total bilirubin], the Simplified Acute Physiology Score (SAPS) II, the Sequential Organ Failure Assessment (SOFA) score, and the Model for End-Stage Liver Disease (MELD) score. For laboratory events, the initial value measurement after admission and before surgery was used for further analyses. Normal values of ALB were defined as 3.5–5.5 g/dl, ALT as 5–40 U/L, AST as 8–40 U/L, and total bilirubin as 0.3–1.3 mg/dl. Considering the ratio of <1.5% missing data for each variable, we directly omitted them in further analysis.

### Outcome Variables

We included the following outcome variables in the study: length of hospital stay, length of ICU stay, hospital mortality, 90-day mortality (post-ICU admission), and 4-year mortality. Among them, hospital mortality was chosen as the primary endpoint of the study, and 4-year mortality was chosen as the secondary endpoint. If a patient was admitted to the ICU multiple times during a single hospitalization, the total ICU hospitalization time was calculated into the length of ICU stay. Because only patients in the CareVue system were followed for at least 4 years, the present study analyzed their 4-year mortality in the CareVue system.

### Statistical Analysis

Continuous variables are presented as the median (interquartile range) or mean ± SD and were compared by Mann-Whitney *U*-test or *t*-test. Categorical data are presented as numbers with proportions and were analyzed by the χ^2^ test. The correlation between the length of ICU stay and hospital stay with laboratory parameters was analyzed by using the non-parametric Spearman's rank correlation test. Survival curves were estimated using the Kaplan-Meier method and compared by the log-rank test. Logistic regression was applied for the uni- or multi-variate analyses to identify independent prognostic factors for mortality (hospital, 90-day, and 4-year mortality) for cardiac surgery patients without liver disease. Two different models were used for potential confounders adjusting: Model 1 was adjusted for SOFA score, creatinine, SpO_2_, hypertension, congestive heart failure, and chronic pulmonary disease. Model 2 was further adjusted for height, SBP, and SAPS II score. *p* < 0.05 were considered statistically significant.

## Results

### Baseline Characteristics of the Study Population

A total of 2,565 patients were included in our study, of which 63 patients (2.46%) died in the hospital. The baseline characteristics of the study population are briefly summarized in Table 1, which shows demographics, vital signs, laboratory events, comorbidities, and scores.

**Table 1:** Baseline characteristics of the study population with different survival statuses in hospital.

	**Survivors (*n* = 2,502)**	**Non-survivors (*n* = 63)**	***P*-value**
**Demographics**			
Age	68.20 ± 12.03	70.38 ± 13.11	0.156
Male, *n* (%)	1,669 (66.71%)	40 (63.49%)	0.593
Weight (kg)	81.55 (69.9–94.4)	75 (63.6–91.5)	0.397
Height (cm)	170.18 (162.56–177.8)	167.64 (157.48–177.8)	0.0045
**Vital signs**			
HR, beats/min	84.31 (78.26–90.45)	86 (78.06–95.65)	0.113
SBP, mmHg	112.21 (105.86–119)	109.35 (101.65–114.53)	0.003
DBP, mmHg	56.62 (52.46–61.32)	56.18 (47.79–62.58)	0.304
RR, times/min	17.18 (15.47–19.11)	18.10 (16.03–20.5)	0.022
Temperature, °C	36.81 (36.48–37.17)	36.53 (36.22–36.8)	0.000
SpO_2_, %	98.09 (97.06–98.91)	97.86 (95.38–98.61)	0.000
**Laboratory events**			
WBC, 10^9^/L	12 (9–15.5)	13 (9.2–17)	0.930
Serum sodium, mmol/L	136 (134–138)	137 (134–139)	0.198
Serum potassium, mmol/L	4 (4–5.1)	4.3 (3.9–5.2)	0.976
Glucose, mg/dL	134 (114–161)	129 (112–165)	0.072
Platelets, 10^9^/L	154 (121–201)	176 (104–226)	0.459
BUN, mg/Dl	16 (12–21)	21 (17–33)	0.000
Creatinine, mg/Dl	0.9 (0.7–1.1)	1.2 (0.9–1.6)	0.000
ALB, g/dL	3.8 (3.5–4.1)	3.4 (3.1–3.7)	0.000
ALT, IU/L	22 (16–33)	29 (19–75)	0.000
AST, IU/L	26 (20–41)	60 (21–139)	0.000
Total bilirubin, mg/dL	0.6 (0.4–0.8)	0.7 (0.4–1.2)	0.000
**Comorbidities**			
Congestive heart failure	863(34.49%)	33 (52.38%)	0.003
Cardiac arrhythmias	1463 (58.47%)	44 (69.84%)	0.070
Valvular disease	659 (26.34%)	19 (30.16%)	0.497
Pulmonary circulation disorder	250 (9.99%)	10 (15.87%)	0.127
Peripheral vascular	362 (14.47%)	14 (22.22%)	0.086
Hypertension	1780 (71.14%)	33 (52.38%)	0.001
Chronic pulmonary	530 (21.18%)	20 (31.75%)	0.044
Uncomplicated diabetes	673 (26.90%)	15 (23.81%)	0.585
Complicated diabetes	166 (6.63%)	7 (11.11%)	0.162
Renal failure	285 (11.39%)	13 (20.64%)	0.024
**Scores**			
SAPS II	36 (30–43)	45 (33–57)	0.000
SOFA	5 (3–7)	8 (4–11)	0.000
MELD	12.9 (10–16.65)	18 (11–23.78)	0.000

*Values are presented as the mean ± standard deviation, median (interquartile range), or the number of patients (%)*.

*ALB, blood albumin; ALT, blood alanine transaminase; AST, blood aspartate transaminase; BUN, blood urea nitrogen; HR, heart rate; SBP, systolic blood pressure; DBP, diastolic blood pressure; RR, respiratory rate; SpO_2_, percutaneous oxygen saturation; WBC, white blood cell; SAPS II, Simplified Acute Physiology Score II; SOFA, Sequential Organ Failure Assessment. MELD, Model end-stage liver disease*.

The demographic characteristics of the study population are shown in Table 1. No significant difference was observed in age, sex, or weight between non-survivors and survivors. Non-survivors had much lower ALB values (3.8 vs. 3.4, *p* = 0.000) and higher ALT (22 vs. 29, *p* = 0.000), AST (26 vs. 60, *p* = 0.000), and total bilirubin (0.6 vs. 0.7, *p* = 0.000) levels (Table 1). Non-survivors tended to have a shorter height, lower SBP, temperature, and SpO_2_ values, and higher RR, creatinine, BUN, SAPS II scores, and SOFA scores, as well as a history of congestive heart failure, hypertension, chronic pulmonary disease, and renal failure (Table 1).

### The Prognostic Significance of the Liver Function Index for Cardiac Surgery Patients Without Liver Disease

We used Spearman's rank correlation test to investigate the association between the liver function index and the length of hospital stay and ICU stay in cardiac surgery patients without liver disease, and the results are briefly summarized in Table 2. ALB was significantly negatively associated with length of hospital stay and ICU stay (hospital stay: Spearman's rho = −0.297, *p* = 0.000; ICU stay: Spearman's rho = −0.293, *p* = 0.000). ALT, AST, and total bilirubin were significantly positively associated with length of hospital stay and ICU stay (for ALT, hospital stay: Spearman's rho = 0.082, *p* = 0.000; ICU stay: Spearman's rho = 0.069, *p* = 0.001; for AST, hospital stay: Spearman's rho = 0.096, *p* = 0.000; ICU stay: Spearman's rho = 0.214, *p* = 0.000; for total bilirubin, hospital stay: Spearman's rho = 0.099, *p* = 0.000; ICU stay: Spearman's rho = 0.156, *p* = 0.000).

**Table 2:** The correlation of blood albumin (ALB), alanine transaminase (ALT), aspartate transaminase (AST), and total bilirubin with hospital stay and ICU stay.

	**Length of hospital stay**		**Length of ICU stay**	
	**Spearman's Rho**	***P*-value**	**Spearman's Rho**	***P*-value**
ALB, g/dL	−0.297	0.000	−0.293	0.000
ALT, IU/L	0.082	0.000	0.069	0.001
AST, IU/L	0.096	0.000	0.214	0.000
Total bilirubin, mg/dL	0.099	0.000	0.156	0.000

Next, the correlation of the liver function index with hospital mortality of cardiac surgery patients without liver disease was investigated. Quartiles of ALB, ALT, AST, and total bilirubin were significantly correlated with hospital mortality (ALB: *p* = 0.000; ALT: *p* = 0.005; AST: *p* = 0.000; total bilirubin: *p* = 0.005) (Table 3). For ALB, a higher rate of hospital mortality was observed in the first quartile, while for ALT, AST, and total bilirubin, a higher rate of hospital mortality was observed in the fourth quartile. Similar trends were observed when grouping by clinical normal values. For ALB, a higher rate of hospital mortality was observed in the group with a lower value than normal, while for ALT, AST, and total bilirubin, a higher rate of hospital mortality was observed in the group with a higher value than normal (ALB: *p* = 0.000; ALT: *p* = 0.000; AST: *p* = 0.000; total bilirubin: *p* = 0.002) (Table 3).

**Table 3:** The relationship between ALB, ALT, AST, and total bilirubin with hospital mortality.

	**Q1**	**Q2**	**Q3**	**Q4**	***P*-value**	**Lower**	**Normal**	**Higher**	***P*-value**
**ALB**									
Survivors	751 (95.06%)	563 (98.25%)	592 (99.16%)	596 (98.51%)	0.000	594 (94.44%)	1,908 (98.55%)	0 (0%)	0.000
Non-survivors	39 (4.94%)	10 (1.75%)	5 (0.84%)	9 (1.49%)		35 (5.56%)	28 (1.45%)	0 (0%)	
**ALT**									
Survivors	830 (98.57%)	509 (97.88%)	563 (97.74%)	600 (95.69%)	0.005	12 (100.00%)	2,048 (98.18%)	442 (94.65%)	0.000
Non-survivors	12 (1.43%)	11 (2.26%)	13 (2.26%)	27 (4.31%)		0 (0%)	38 (1.82%)	25 (5.35%)	
**AST**									
Survivors	795 (98.15%)	497 (99.00%)	614 (23.94%)	596 (94.45%)	0.000	5 (100.00%)	1,856 (98.62%)	641 (94.54%)	0.000
Non-survivors	15 (1.85%)	5 (1.00%)	8 (0.31%)	35 (5.54%)		0 (0%)	26 (1.38%)	37 (5.46%)	
**Total bilirubin**									
Survivors	1,290 (98.25%)	363 (98.11%)	228 (97.85%)	621 (95.69%)	0.005	129 (99.23%)	2,116 (97.83%)	257 (94.49%)	0.002
Non-survivors	23 (1.75%)	7 (1.89%)	5 (2.15%)	28 (4.31%)		1 (0.77%)	47 (2.17%)	15 (5.51%)	

*Q, Quartile of serum ALB, ALT, AST and total bilirubin value*.

*Lower, lower than clinical normal value*.

*Normal, clinical normal value*.

*Higher, higher than clinical normal value*.

Then, the correlation of the liver function index with a 90-day mortality of cardiac surgery patients without liver disease was investigated. As shown in Table 4, for ALB, a higher rate of 90-day mortality was observed in the first quartile, and for ALT and AST, a higher rate of 90-day mortality was observed in the fourth quartile (ALB: *p* = 0.000; ALT: *p* = 0.043; AST: *p* = 0.000). For total bilirubin, the 90-day mortality of the third and fourth quartiles was higher than that of the first and second quartiles (*p* = 0.000). Interestingly, grouping according to the clinical normal value revealed different trends than quartile grouping. For ALB and AST, a higher rate of 90-day mortality was observed in the group with lower and higher values than normal, while for total bilirubin, a higher rate of 90-day mortality was observed in the group with a higher value (ALB: *p* = 0.000; ALT: *p* = 0.002; AST: *p* = 0.000; total bilirubin: *p* = 0.021).

**Table 4:** The relationship between ALB, ALT, AST, and total bilirubin with 90-day mortality.

	**Q1**	**Q2**	**Q3**	**Q4**	***P*-value**	**Lower**	**Normal**	**Higher**	***P*-value**
**ALB**									
Survivors	721 (91.27%)	554 (96.68%)	582 (97.49%)	585 (96.69%)	0.000	572 (90.94%)	1,870 (96.59%)	0 (0%)	0.000
Non-survivors	69 (8.73%)	19 (3.32%)	15 (2.51%)	20 (3.31%)		57 (9.06%)	66 (3.41%)	0 (0%)	
**ALT**									
Survivors	802 (95.25%)	503 (96.73%)	552 (95.83%)	585 (93.30%)	0.043	11 (91.67%)	2,001 (95.93%)	430 (92.08%)	0.0002
Non-survivors	40 (4.75%)	17 (3.27%)	24 (4.17%)	42 (6.70%)		1 (8.33%)	85 (4.07%)	37 (7.92%)	
**AST**									
Survivors	775 (95.68%)	487 (97.01%)	602 (96.78%)	578 (91.60%)	0.000	4 (80.00%)	1,816 (96.49%)	622 (91.74%)	0.000
Non-survivors	35 (4.32%)	15 (2.99%)	20 (3.22%)	53 (8.40%)		1 (20.00%)	66 (3.51%)	56 (8.26%)	
**Total bilirubin**									
Survivors	1,265 (96.34%)	359 (97.03%)	217 (93.13%)	601 (92.60%)	0.000	126 (96.92%)	2,066 (95.52%)	250 (91.91%)	0.021
Non-survivors	48 (3.66%)	11 (2.97%)	16 (6.87%)	48 (7.40%)		4 (3.08%)	97 (4.48%)	22 (8.09%)	

*Q, Quartile of serum ALB, ALT, AST and total bilirubin value*.

*Lower, lower than clinical normal value*.

*Normal, clinical normal value*.

*Higher, higher than clinical normal value*.

For 4-year mortality, only patients in CareVue who were followed for at least 4 years were analyzed. As shown in Table 5, for ALB, higher 4-year mortality was observed in the first quartile and in the group with lower values than normal (all *p* = 0.000). For AST, higher 4-year mortality was observed in the first and fourth quartiles (*p* = 0.023), and in the group with lower and higher values than normal (*p* = 0.007). For total bilirubin, higher 4-year mortality was observed in the third and fourth quartiles and in the group with lower and higher values than normal (all *p* = 0.000).

**Table 5:** The relationship between ALB, ALT, AST, and total bilirubin with 4-year mortality.

	**Q1**	**Q2**	**Q3**	**Q4**	***P-*value**	**Lower**	**Normal**	**Higher**	***P*-value**
**ALB**									
Survivors	356 (71.15%)	305 (83.11%)	300 (86.46%)	267 (88.41%)	0.000	276 (67.64%)	952 (84.92%)	0 (0%)	0.000
Non-survivors	157 (28.85%)	62 (16.89%)	47 (13.54%)	35 (11.59%)		132 (32.35%)	169 (15.08%)	0 (0%)	
**ALT**									
Survivors	381 (79.21%)	252 (83.17%)	270 (79.18%)	325 (80.44%)	0.529	8 (88.89%)	985 (80.61%)	235 (78.86%)	0.643
Non-survivors	100 (20.79%)	51 (16.83%)	71 (20.82%)	79 (19.56%)		1 (11.11%)	237 (19.39%)	63 (21.14%)	
**AST**									
Survivors	415 (81.69%)	231 (15.11%)	282 (18.44%)	300 (75.38%)	0.023	2 (66.67%)	907 (82.30%)	319 (75.24%)	0.007
Non-survivors	93 (18.31%)	56 (3.66%)	54 (3.53%)	98 (24.62%)		1 (33.33%)	195 (17.70%)	105 (24.76%)	
**Total bilirubin**									
Survivors	644 (83.10%)	179 (84.83%)	108 (77.70%)	297 (73.51%)	0.000	54 (77.14%)	1,058 (81.95%)	116 (69.04%)	0.000
Non-survivors	131 (16.90%)	32 (15.17%)	31 (22.30%)	107 (26.49%)		16 (22.86%)	233 (18.04%)	52 (30.95%)	

*Q, Quartile of serum ALB, ALT, AST and total bilirubin value*.

*Lower, lower than clinical normal value*.

*Normal, clinical normal value*.

*Higher, higher than clinical normal value*.

**Figure 1:**
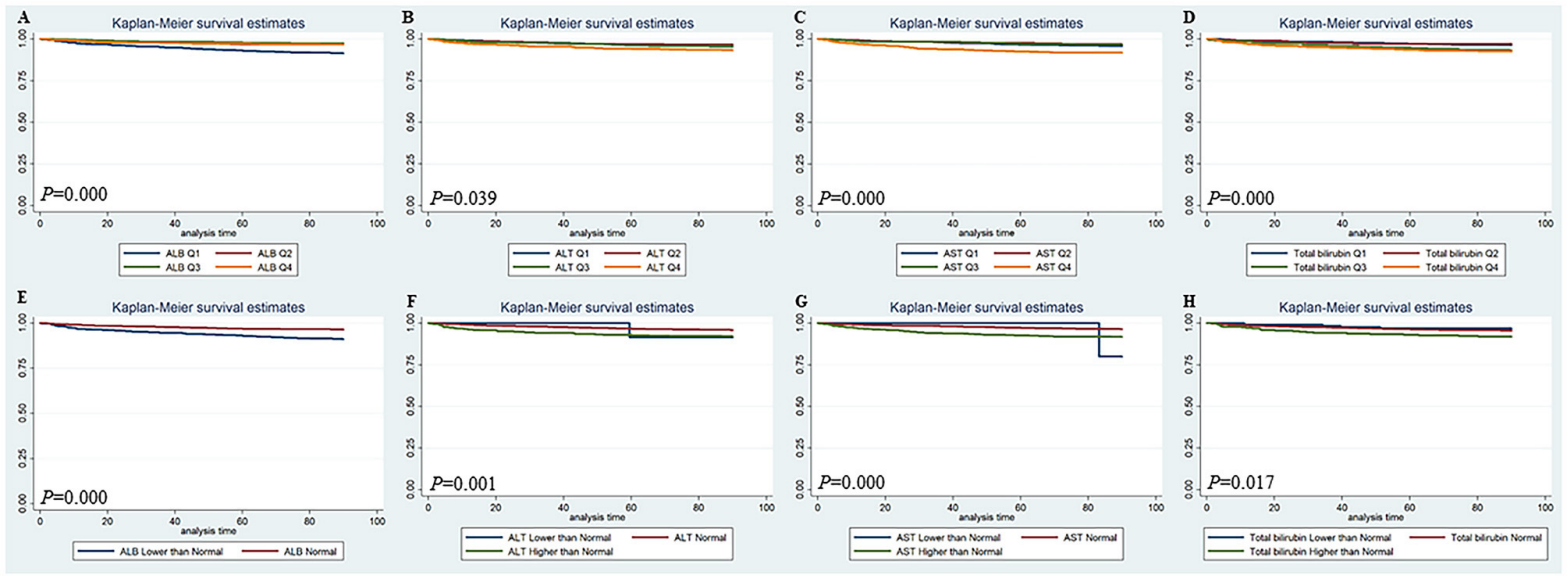
Kaplan-Meier 90-day survival curves comparing patients by liver function index levels. Q, Quartile of serum ALB, ALT, AST and total bilirubin value; Normal, clinical normal value; ALB, blood albumin; ALT, blood alanine transaminase; AST, blood aspartate transaminase.

**Figure 2:**
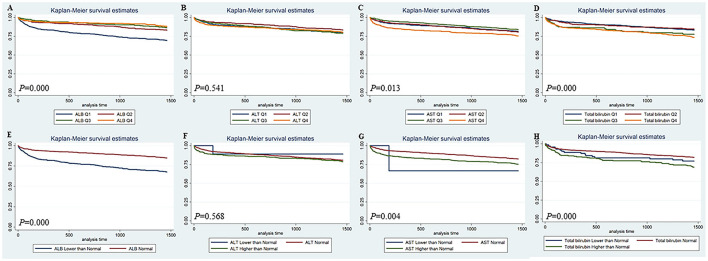
Kaplan-Meier 4-year survival curves comparing patients by liver function index levels. Q, Quartile of serum ALB, ALT, AST and total bilirubin value; Normal, clinical normal value; ALB, blood albumin; ALT, blood alanine transaminase; AST, blood aspartate transaminase.

The Kaplan-Meier survival curves comparing patients by liver function index levels are shown in Figure 1. The results showed that patients in the first quartile of ALB and in the fourth quartile of ALT, AST, and total bilirubin had the lowest 90-day survival (all *p* < 0.05) (Figures 1A–D). The patients with lower ALB, lower and higher ALT/AST, and higher total bilirubin had lower 90-day survival (all *p* < 0.05) (Figures 1E–H). Moreover, patients in the first quartile of ALB, in the fourth quartile of AST, and in the third and fourth quartiles of total bilirubin had lower 4-year survival (all *p* < 0.05), but the quartiles of ALT were not significantly different (Figures 2A–D). The patients with lower ALB and lower and higher AST/total bilirubin had lower 4-year survival (all *p* < 0.05), but such differences in ALT levels were not observed (Figures 2E–H).

A univariate logistic regression analysis was performed. As shown in Table 6, ALB was associated with hospital mortality (OR = 0.36, 95% CI = 0.25–0.52, *p* = 0.000), 90-day mortality (OR = 0.37, 95% CI = 0.28–0.49, *p* = 0.000), and 4-year mortality (OR = 0.41, 95% CI = 0.33–0.50, *p* = 0.000). ALT was associated with hospital mortality (OR = 1.00, 95% CI = 1.00–1.01, *p* = 0.000) and 90-day mortality (OR = 1.00, 95% CI = 1.00–1.01, *p* = 0.001) but not 4-year mortality (OR = 1.00, 95% CI = 0.99–1.00, *p* = 0.214). AST was associated with hospital (OR = 1.00, 95% CI = 1.00–1.01, *p* = 0.000) and 90-day mortality (OR = 1.00, 95% CI = 1.00–1.01, *p* = 0.001) but not 4-year mortality (OR = 1.00, 95% CI = 0.99–1.00, *p* = 0.079). Total bilirubin was associated with hospital mortality (OR = 1.49, 95% CI = 1.23–1.81, *p* = 0.000), 90-day mortality (OR = 1.41, 95% CI = 1.20–1.66, *p* = 0.000), and 4-year mortality (OR = 1.46, 95% CI = 1.24–1.73, *p* = 0.000).

**Table 6:** Univariate logistic regression analyses for prognosis in cardiac surgery patients without liver disease.

	**Hospital mortality**		**90-day mortality**		**4-year mortality**	
**Variable**	**OR (95% CI)**	***P***	**OR (95% CI)**	**P**	**OR (95% CI)**	***P***
ALB, g/dL	0.36 (0.25–0.52)	0.000	0.37 (0.28–0.49)	0.000	0.41 (0.33–0.50)	0.000
ALT, IU/L	1.00 (1.00–1.01)	0.000	1.00 (1.00–1.01)	0.001	1.00 (0.99–1.00)	0.214
AST, IU/L	1.00 (1.00–1.01)	0.000	1.00 (1.00–1.01)	0.001	1.00 (0.99–1.00)	0.079
Total bilirubin, mg/dL	1.49 (1.23–1.81)	0.000	1.41 (1.20–1.66)	0.000	1.46 (1.24–1.73)	0.000

*For 4-year mortality, only patients in the CareVue system who were followed for at least 4-year were analyzed*.

The results of multivariate analysis are shown in Table 7. For multivariate analysis, Model 1 was adjusted for SOFA score, creatinine, SpO_2_, hypertension, congestive heart failure, and chronic pulmonary disease. Model 2 was further adjusted for height, SBP, and SAPS II score. ALB was associated with hospital mortality (Model 1: OR = 0.48, 95% CI = 0.33–0.70, *p* = 0.000; Model 2: OR = 0.43, 95% CI = 0.29–0.62, *p* = 0.000), 90-day mortality (Model 1: OR = 0.46, 95% CI = 0.35–0.61, *p* = 0.000; Model 2: OR = 0.42, 95% CI = 0.31–0.55, *p* = 0.000), and 4-year mortality (Model 1: OR = 0.47, 95% CI = 0.37–0.58, *p* = 0.000; Model 2: OR = 0.48, 95% CI = 0.38–0.59, *p* = 0.000) in both models. ALT was associated with hospital mortality (Model 1: OR = 1.00, 95% CI = 1.00–1.01, *p* = 0.003; Model 2: OR = 1.00, 95% CI = 1.00–1.01, *p* = 0.004) and 90-day mortality (Model 1: OR = 1.00, 95% CI = 1.00–1.01, *p* = 0.015; Model 2: OR = 1.00, 95% CI = 1.00–1.01, *p* = 0.008) in both models, but not 4-year mortality (Model 1: OR = 1.00, 95% CI = 0.99–1.19, *p* = 0.793; Model 2: OR = 1.00, 95% CI = 0.99–1.00, *p* = 0.647). AST was associated with hospital mortality (Model 1: OR = 1.00, 95% CI = 1.00–1.01, *p* = 0.002; Model 2: OR = 1.00, 95% CI = 1.00–1.01, *p* = 0.005) and 90-day mortality (Model 1: OR = 1.00, 95% CI = 1.00–1.01, *p* = 0.007; Model 2: OR = 1.00, 95% CI = 1.00–1.01, *p* = 0.008) in both models, but not 4-year mortality (Model 1: OR = 1.00, 95% CI = 0.99–1.00, *p* = 0.286; Model 2: OR = 1.00, 95% CI = 0.99–1.00, *p* = 0.304). Total bilirubin was associated with hospital mortality (Model 1: OR = 1.33, 95% CI = 1.07–1.65, *p* = 0.010; Model 2: OR = 1.45, 95% CI = 1.19–1.78, *p* = 0.000), 90-day mortality (Model 1: OR = 1.29, 95% CI = 1.07–1.54, *p* = 0.006; Model 2: OR = 1.40, 95% CI = 1.18–1.65, *p* = 0.000), and 4-year mortality (Model 1: OR = 1.37, 95% CI = 1.15–1.64, *p* = 0.001; Model 2: OR = 1.48, 95% CI = 1.23–1.77, *p* = 0.000) in both models (Table 7).

**Table 7:** Association between ALB, ALT, AST, and total bilirubin with prognosis of cardiac surgery patients without liver disease.

**Outcome**	**OR**	**95% CI**	***P*-value**
**Hospital mortality**				
ALB	Model 1	0.48	0.33–0.70	0.000
	Model 2	0.43	0.29–0.62	0.000
ALT	Model 1	1.00	1.00–1.01	0.003
	Model 2	1.00	1.00–1.01	0.004
AST	Model 1	1.00	1.00–1.01	0.002
	Model 2	1.00	1.00–1.01	0.005
Total bilirubin	Model 1	1.33	1.07–1.65	0.010
	Model 2	1.45	1.19–1.78	0.000
**90-day mortality**				
ALB	Model 1	0.46	0.35–0.61	0.000
	Model 2	0.42	0.31–0.55	0.000
ALT	Model 1	1.00	1.00–1.01	0.015
	Model 2	1.00	1.00–1.01	0.008
AST	Model 1	1.00	1.00–1.01	0.007
	Model 2	1.00	1.00–1.01	0.008
Total bilirubin	Model 1	1.29	1.07–1.54	0.006
	Model 2	1.40	1.18–1.65	0.000
**4-year mortality**				
ALB	Model 1	0.47	0.37–0.58	0.000
	Model 2	0.48	0.38–0.59	0.000
ALT	Model 1	1.00	0.99–1.19	0.793
	Model 2	1.00	0.99–1.00	0.647
AST	Model 1	1.00	0.99–1.00	0.286
	Model 2	1.00	0.99–1.00	0.304
Total bilirubin	Model 1	1.37	1.15–1.64	0.001
	Model 2	1.48	1.23–1.77	0.000

*Model 1 was adjusted for SOFA score, creatinine, SpO_2_, hypertension, congestive heart failure, and chronic pulmonary*.

*Model 2 was adjusted for height, SBP, and SAPS II score*.

*For 4-year mortality, only patients in the CareVue system who were followed for at least 4-year were analyzed*.

## Discussion

There is a complex pathophysiological interaction between the heart and the liver. Thus, pathological changes in one organ system can cause structural and functional abnormalities in the other organ systems (9). In heart failure patients, liver dysfunction is a well-known complication, and it is a risk factor for not only mortality in heart failure patients but also outcomes of cardiac surgery patients (5, 10, 11). However, similar outcome data are lacking in cardiac surgery patients without a history of acute hepatic failure or primary significant liver disease. Our data showed that abnormal ALB, AST, ALT, and total bilirubin were associated with the length of hospital stay and ICU stay, hospital mortality, and 90-day and 4-year mortality in cardiac surgery patients without liver disease (**Tables 2–6**, Figures 1, 2). Abnormal ALB, AST, ALT, and total bilirubin were independent risk factors for hospital mortality and 90-day mortality, but only ALB and total bilirubin were independent risk factors for 4-year mortality (Table 7). Our investigation suggested that preoperative LFT abnormalities were associated with short-term and long-term prognosis in cardiac surgery patients without liver disease.

Low serum ALB concentration or hypoalbuminemia was considered to be an indicator of prognosis after cardiovascular surgery and might be induced by congestive heart failure or chronic liver insufficiency (12–14). It is well-known that serum ALB can help to maintain the intravascular volume, partially by facilitating the integrity of the vasculature (15). Thus, a lower serum ALB level might result in more tissue edema and reduce the circulating volume by extravasation. In our study, preoperative low serum ALB was significantly associated with short-term and long-term prognosis in cardiac surgery patients without liver disease (Tables 2–6, Figures 1, 2) and could serve as an independent risk factor for mortality (Table 7). We found that the albumin level of the non-survivors was significantly lower than that of survivors although the difference is quite small (3.4 vs. 3.8 mg/dl). This may indicate that, when the albumin level is lower than the normal level, even a slight decrease in albumin level will increase the risk of death of the patient. Thus, preoperative nutritional repletion to correct hypoalbuminemia can benefit cardiac patients before cardiac surgery and avert possible deleterious effects in the postoperative period.

High serum total bilirubin could be induced by haemolysis, decreased liver perfusion, and chronically low cardiac output, or hepatocyte dysfunction due to liver congestion in right-sided heart failure (11, 16, 17). The preoperative level of serum bilirubin was an independent risk factor for cardiac surgery in patients with liver cirrhosis (18). Our results indicated that preoperative high serum total bilirubin could serve as an independent risk factor for short-term and long-term prognosis in cardiac surgery patients without liver disease (Tables 2–7, Figures 1, 2). High serum total bilirubin in cardiac surgery patients without liver disease might be caused by hepatic hypoperfusion and/or hepatic congestion. Under these circumstances, optimizing the treatment of congestive heart failure before surgery can reduce the preoperative serum total bilirubin level. Therefore, the outcome of cardiac surgery could be improved. However, to investigate this problem, more research needs to be done, as a conclusion cannot be drawn from our data.

Alanine transaminase and AST are classic cytoplasmic enzymes and inflammation-associated components that can be immediately released into the bloodstream following the deleterious effect of heart failure on the liver (19, 20). For patients with heart failure, there is a graded relationship between admission transaminase levels and surrogate indicators of in-hospital mortality (21). Furthermore, more significant ALT elevation can be used as an independent predictor of hospital mortality, deterioration of renal function, ICU admission, and longer time of hospital stay (21). Preoperative and postoperative AST/ALT might be an independent predictor of AKI after cardiac surgery (20). Our research is consistent with previous reports. ALT and AST were found to be independent risk factors of hospital mortality and 90-day mortality in cardiac surgery patients without liver disease but not 4-year mortality (Tables 2–7, Figures 1, 2). Notably, increased transaminase levels in patients with significantly increased mortality may be caused by progressive heart pump failure. This hypothesis is supported by the high proportion of positive inotropic and/or vasopressor support in this subgroup of patients and the lack of association between ALT and fatal arrhythmias (21).

## Limitations

As a single-center retrospective study, this study has all the limitations of a retrospective observational study. Our findings need to be further confirmed in a larger multicentered cohort or a randomized controlled trial. In addition, the numbers of survivors and non-survivors are quite different, which may cause slight bias in the study.

## Conclusion

In summary, we demonstrated that abnormal LFTs were significantly correlated with the length of hospital stay and ICU stay of cardiac surgery patients without liver disease. Abnormal LFTs were associated with higher hospital mortality, 90-day mortality, and 4-year mortality, as well as lower 90-day and 4-year survival. LFTs could serve as an independent predictor of the hospital, 90-day and 4-year mortality in cardiac patients without liver disease.

## Data Availability Statement

Publicly available datasets were analyzed in this study. This data can be found at the Medical Information Mart for Intensive Care III (MIMIC-III): https://mimic.mit.edu/ and https://physionet.org/.

## Ethics Statement

The studies involving human participants were reviewed and approved by Institutional Review Boards of the Massachusetts Institute of Technology (Cambridge, MA, USA) (Record ID 41268972). Written informed consent for participation was not required for this study in accordance with the national legislation and the institutional requirements.

## Author Contributions

ZW, JY, and JH conceived and designed the study. LS, YA, LinL, LihL, YZ, and JH provided, selected, assembled, analyzed, and interpreted the data. All authors contributed to data analysis, drafting, critically revising the paper, agreed to be accountable for all aspects of the work, and read and confirmed that they meet ICMJE criteria for authorship.

## Funding

This study was funded by the National Natural Science Foundation of China (81900294, 81770319, 81570039, and 82070297) and the Natural Science Foundation of Hunan Province, China (2018JJ2369, 2018JJ2362).

## Conflict of Interest

The authors declare that the research was conducted in the absence of any commercial or financial relationships that could be construed as a potential conflict of interest.

## Publisher's Note

All claims expressed in this article are solely those of the authors and do not necessarily represent those of their affiliated organizations, or those of the publisher, the editors and the reviewers. Any product that may be evaluated in this article, or claim that may be made by its manufacturer, is not guaranteed or endorsed by the publisher.

## Abbreviations

ALB: blood albumin
ALT: blood alanine transaminase
AST: blood aspartate transaminase
BIDMC: Beth Israel Deaconess Medical Center
CPB: cardiopulmonary bypass
DBP: diastolic blood pressure
CABG: coronary artery bypass grafting
HR: heart rate
ICU: intensive care unit
LFTs: liver function tests
MIT: Massachusetts Institute of Technology
MELD: Model for End-Stage Liver Disease score
MIMIC III: Multiparameter Intelligent Monitoring in Intensive Care III database
RR: respiratory rate
SAPS II: Simplified Acute Physiology Score II
SBP: systolic blood pressure
SOFA: Sequential Organ Failure Assessment score
SpO_2_: percutaneous oxygen saturation
SQL: Structured Query Language
WBC: white blood cell.
